# Tobacco

**Published:** 1874-12

**Authors:** 


### TOBACCO.

Two hundred and sixty million pounds are raised in the United States in one year, six millions of which are produced in Hartford County, Connecticut, the best of which is the best in the world, is sent to Hamburg and Havana, made up into cigars, and sold as the pure Cuban article.

Tobacco, Tea and Spirits are used all over the world to soothe and to cheer troubled minds and wearied bodies. That they do these things is undeniable. To what extent they should be used for these purposes is a question which is variously answered. Those who have never employed them for these purposes know nothing about it, are very generally ready to disparage the practice, and are quite impatient with those who indulge. Their employment is always dangerous, because the tendencies are to an excess, which injures the body and impairs the mind. The safe side is the best—never touch them. Whether any one is justifiable in voluntarily exposing himself to possibly terrible evils is the question.

